# Reasons for resistance to change in nursing: an integrative review

**DOI:** 10.1186/s12912-023-01460-0

**Published:** 2023-09-11

**Authors:** Rozita Cheraghi, Hossein Ebrahimi, Nasrin Kheibar, Mohammad Hasan Sahebihagh

**Affiliations:** 1 Ph.D. Candidate in Nursing. Student Research Committee, Tabriz University of medical sciences, Tabriz, Iran; 2 Department of Psychology Nursing, School of Nursing and Midwifery, Tabriz University of Medical Sciences, Tabriz, Iran; 3 Community Health Nursing Department, School of Nursing and Midwifery, Tabriz University of medical sciences, Tabriz, Iran

**Keywords:** Resistance, Change, Nursing, Integrated review

## Abstract

**Background:**

Change is a very complex and multifaceted phenomenon that is intertwined with the understanding of nursing practice, so, resistance to change in nursing can be considered as an important challenge. Knowing the reasons for this resistance can help in solving it in nursing. Therefore, the present study was conducted with the aim of investigating the reasons for resistance to change in nursing as an integrated review.

**Methods:**

This integrative review was conducted using the Whittemore & Knafl method in 5 stages, including problem identification, searching the literature, evaluating primary sources, analyzing data, and presenting the results. Databases like SID, Irandoc, Magiran, Google Scholar, Web of Science, PubMed, CINAHL, and Scopus were searched using the keywords; “Resistance”, “Change”, “Nursing”, “Resistance to Change” and their Persian equivalents in the time range of 2000 to January 2023. After applying inclusion criteria and assessing the articles using Bowling’s Quality Assessment Tool, finally, 15 papers were included from 2964.

**Results:**

After reviewing and critically appraisal of the qualified articles, the findings were placed in three main categories including; (1) individual factors, (2) interpersonal factors, and (3) organizational factors and six subcategories.

**Conclusion:**

Undoubtedly, change is an integral component in nursing care, and resistance to it is the result of a set of individual, interpersonal and organizational factors that change managers should pay special attention to in order to make changes due to the reasons of this resistance, and the development process of developing changes in the clinical field is easily possible.

## Introduction

Change is a very complex and multifaceted phenomenon that is intertwined with the understanding of nursing practice [1, 2], and it’s happening fast in health care, so, all nurses, as a part of the change process, must be knowledgeable and skilled [3]. In the dynamic environment of healthcare, the organization’s agility to change is the key to its survival [4]. However, not all employees in an organization react equally to ongoing changes in their workplace organization, some employees respond to these changes with enthusiasm and as opportunities for learning and growth, while others resist such changes and show increasing feelings of frustration, alienation, and sadness [5]. It would be said; a key barrier to implementing change is employee resistance to change [6]. So that, resistance to change (RtC) is widely recognized as the main reason for failure when it comes to change initiatives. Despite the importance of this issue; still relatively limited knowledge exist about the factors that cause resistance to change in the organization [7].

Generally defining the concept of “resistance to change” is not easy [8], but based on the literature; resistance is defined as the informal and covert behavior of an individual in response to a perceived or actual threat to maintain the status quo [9, 10]. In other words, resistance is defined as failure to do anything that is asked by managers from employees [11]. Also, behavioral resistance is known as a prevent or stop change [9], which can ultimately be the main cause of change failure [12]. However, sometimes the nature of resistance can finally be a valuable resource for achieving change [8, 13].

Organizational resistance can be caused by power and conflict, or be the result of differences in functional orientation, structure, and organizational culture [14]. Some of the reasons for the organizational changes according to the studies are restructuring in the workplace, advances in technology, a greater need for efficiency, and the growth of new services [3]. Some others at the group level include resistance to change caused by group norms, group cohesion, group thinking, and intensifying commitment [15], and at the individual level include uncertainty and Lack of job security, selective perception and retention, and getting used to the current work [16].

Implementing change in the healthcare system is difficult, challenging, and often has short-term results [17], especially when the context of change includes changes in care organization, modification of common clinical practices, increased collaboration between different disciplines, and changes in patient behavior [18]. This happens because the healthcare services are delivered in an environment where groups of people act in different and unpredictable ways, where tensions arise through opposing, competing, or collaborative forces, and where decisions are influenced by priorities, and records of healthcare professionals are adopted [19].

Studies show that; Nurses are inherently resistant to clinical change [1], and there are several reasons for this. RtC in nursing is likely based on fear, uncertainty, doubt, frustration, distrust, confusion, and anger [20]. Although accepting change is challenging for nurses, resistance is usually an ordinary and predictable reaction to change [21].

Resistance has historically been viewed with negative consequences due to its potential impact on organizational success [9]. However, resistance is a normal response to a threat to the status quo because change requires people to abandon their current processes [22]. Individual’s resistance can be an obstacle to implementing change [23], and plays an important role in successful adaptation to change [22]. Improvements in the changes in the provision of organizational healthcare are often positive and carried out to improve the quality, safety, and efficiency of healthcare, thus increasing the experiences for patients and employees. However, despite these positive results, nurses often face resistance to change and are considered a natural consequence [9].

Accepting change in the core of nursing and health care is considered a challenge, and some of these challenges are related to the movement of information and knowledge from research to the implementation of evidence-based best practices [24]. It is because employees and organizations simply do not like change [25], and the organizational culture (context and environment of the organization) that is conservative and may strengthen the resistance that can prevent the implementation of new changes [26]. This kind of resistance is the result of the cognitive and behavioral reactions of the recipients of the change towards the change [27], which is often in conflict with the organizational identity and causes an unpleasant image of individual, and threats the organizational identity [28]. Although the effect of resistance to change is not static: instead, it can have a negative, festering effect on relationships with perceived organizational effectiveness and commitment to the organization over time [5].

What is obvious is that resistance to change in nursing care can be an important challenge, although various studies have addressed the concept of change, however, very few and scattered studies have focused on the reasons for resistance in nursing. Therefore, this study was conducted with the aim of an integrated overview of the reasons for resistance to change in nurses. An integrative review is a specialized review method that summarizes empirical or theoretical studies that have already been conducted to provide a more comprehensive understanding of a specific phenomenon or healthcare problem [29]. In fact, integrated reviews have the potential to expand the body of knowledge and create nursing science, knowledge of research, practice, and policies, at the same time, this category of studies shows the current state of knowledge in each field, helps to develop theory and has a direct application in practice and health policies [30]. Therefore, the results of this study can help to clarify the reasons for resistance to change in nursing and, as a result, to solve it.

## Methods

### Study design

This study is an integrated review based on articles related to the reasons for resistance to change in nursing which was conducted to collect data from various studies. This integrative review was conducted using the Whittemore & Knafl method in 5 stages of review, including (a) problem identification, (b) searching the literature, (c) evaluating data from primary sources, (d) analyzing data, and (e) presenting the results, using of this method also increases the rigor of this study [30–32].

### Search strategy

Based on the Whittemore & Knafl method, 1) in the first stage, the following question was set to answer the study’s aim: What are “the reasons for resistance to change in nursing”?

2) In the second stage, searching for articles was conducted by two researchers in the time range from 2000 to January 2023. We searched databases such as; Persian database(Magiran, SID, Irandoc), Google Scholar, Web of Science, PubMed, CINAHL, and Scopus by using the keywords; “Resistance”, “Change”, “Nursing”, and “Resistance to Change” in English and Persian separately or combined by using the Boolean operators(AND and OR). In this stage; the results of the comprehensive search included 2964 articles after reviewing them based on the inclusion criteria such as: accessing the full text of the article, including the keywords in the title and abstract of the article, and writing in Persian and English language, finally 2949 were removed, and the 15 articles were included.

### Eligibility criteria

**Fig. 1:**
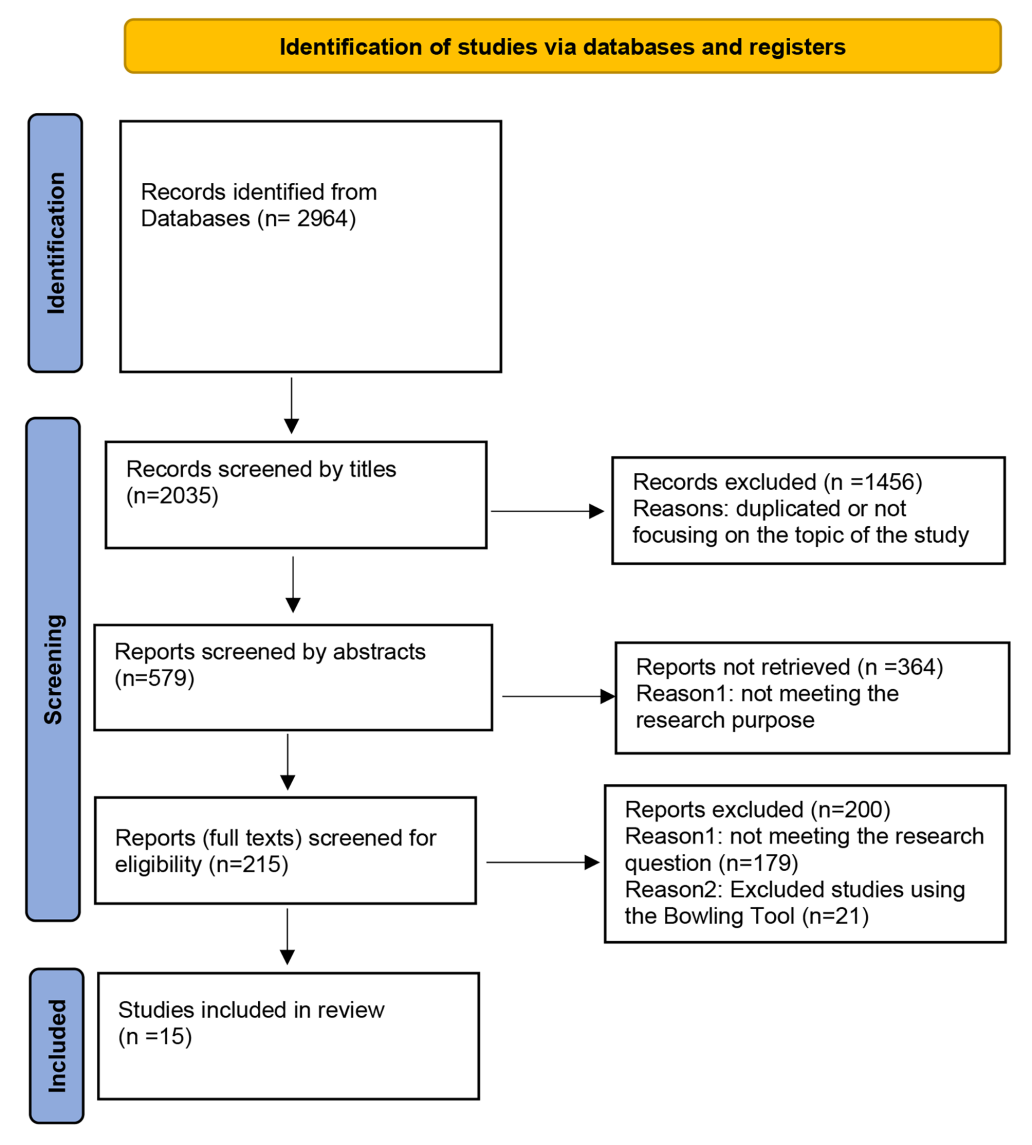
PRISMA flow diagram

3) In this stage, two researchers evaluated the data and the content of selected studies for their quality by using “Bowling’s Quality Assessment Tool“(consists of items for checking the structure of the methodology and presenting the results of the studies: high, moderate, and low-quality) [33], which caused the removal of 5 of these articles by that. Then we compared the results, and finally 15 articles were included in this study (Fig. 1 following the renewed PRISMA guideline 2020) [34].

### Data extraction

The data extraction was based on the main data from the 15 articles included: the publication’s year, the language of the study, the main keywords and the methodology of the study, and also the results.

### Data synthesis

4) In the fourth stage, data were evaluated independently by two researchers and updated in a continuous process, after that, the data were analyzed and interpreted. This process was verified by three authors including; documenting the required data through five methodological stages, and analyzing separately by researchers.

All of the extracted data were read by researchers and determined significant items and similar and different data were assessed and examined throughout the data analysis process.

Eventually, confirmation and verification were performed by all authors to ensure that all 15 reviews were thoroughly evaluated in all of the methodological stages and the results were matched by the research questions of the study.

### Quality appraisal

Whittemore and Knafl (2005) state that assessing the quality of the included evidence is not essential in a supplementary review [31]. All studies meeting the inclusion criteria, regardless of their methodological quality, were retained in the review to examine all evidence of the factors that influenced the nursing role implementation in practice settings. Also, Bowling’s quality checklist was used to appraise the articles, which allowed us to evaluate and compare study objectives, design, methods, analysis, results, discussion, and clinical implications [33].

For our review, studies were deemed to be of relatively high quality, and studies that were in the moderate or low-quality range were omitted.

## Results

5) In the last step, the results were obtained according to the framework of 15 articles that were selected. It is necessary to mention that was found no paper in Persian, and all articles included in this study were in English (Table 1).

**Table 1:** Selective qualified articles

Authors and year	Country/ number of samples	Title
Khai Wah Khaw et al. 2022 [27]	Malaysia / 79 studies	Reactions towards organizational change: a systematic literature Review.
Younghee Cho & et al. 2021 [35]	Republic of Korea/ 223 nurses	Factors associated with nurses’ user resistance to change of electronic health record systems.
Eva Ericson-Lidman et al. 2021 [36]	Sweden/ 15 change agents	Change agents’ experiences of implementing a new organizational culture in residential care for older people: A qualitative study.
Briony Marie DuBose et al. 2020 [9]	San Diego	Resistance to Change: A Concept Analysis.
Michelle Cleary & et al. 2019 [24]	Australia	Change Management in Health Care and Mental Health Nursing.
Jiří Mareš 2018 [8]	Hradec Králové	Resistance of health personnel to changes in healthcare.
Catrin Johanssona et al. 2014 [26]	Sweden/ 133 nurses	Culture as a predictor of resistance to change: A study of competing values in a psychiatric nursing context.
Denise A. Tyler et al. 2014 [6]	US/ 64 administrators	Overcoming Resistance to Culture Change: Nursing Home Administrators’ Use of Education, Training, and Communication.
Kim McMillan et al. 2013 [12]	Canada	Nurses Amidst Change: The Concept of Change Fatigue Offers an Alternative Perspective on Organizational Change.
Carey S. Clark 2013 [20]	Augusta, Maine	Resistance to Change in the Nursing Profession: Creative Transdisciplinary Solutions.
Donna J. Munroe et al. 2011 [37]	US/ 400 nursing facility staff	Culture-change training: Nursing facility staff perceptions of culture change.
Jeffrey D. Ford et al. 2010 [38]	US	Stop blaming resistance to change and start using it. Organizational Dynamics.
Jeffrey D. Ford. 2008 [13]	US	Resistance To Change: The Rest Of The Story.
Beverley & Copnell et al. 2006 [1]	Australia/ 12 nurses	Breaking the silence: nurses’ understandings of change in clinical Practice.
Melanie M. Kan et al. 2004 [39]	Australia/ 50 h observation or interview (nurse/ doctors)	Identifying paradox: A grounded theory of leadership in overcoming resistance to change.

After reviewing and evaluating the qualified articles, the findings were classified into three main categories as follows: (1) individual factors, (2) interpersonal factors, and (3) organizational factors and six subcategories (Table 2).

**Table 2:** Main categories and sub-categories extract from the review of selected articles

Main categories	Sub-Categories	Codes
Individual factors	Individual attitude and perception	- Lack of awareness about the benefits of change [35] - Negative attitude toward change [1] - Uncertainty (doubt) [9, 20] - Negative understanding and belief toward change [8, 9, 13, 24, 27, 39] - Feeling insecure [36] - Negative and defensive feelings towards change (fear, worry, frustration, anger) [9, 20, 27, 38] - Confusion [20, 36] - lack of trust (avoidance of alternative ideas) [20] - Fatigue [12] - Feeling threatened [8] - Lack of readiness to accept change [13]
	Personality characteristics	- Low motivation [36, 27] - Culture of change (indifference, inflexibility) [26] - Unfair judgment of change [27] - Low self-confidence [27] - Conservatism [26] - Reluctance to leave previous habits [6, 26]
Interpersonal factors	Communication and cultural factors	- Colleagues’ opinion [35] - Communicating and expressing changes [13, 24, 27] - Human relations (openness, mutual trust, loyalty) [9, 26] - Individual culture [37, 39]
Organizational factors	Management factors	- Desire to strengthen the existing situation [35] - Difficulty applying change [35] - Organizational support [35] - Lack of participatory management and not being involved in the change process [12, 27] - Lack of appreciation and support [27, 36] - Speed of change [27] - Lack of explicit feedback [27] - Lack of proper education and guidance [20, 27, 35]
	Organizational values	- Organizational Culture [6, 24, 26, 27] - Negative organizational perception [26] - Conflict with organizational identity [27]
	Structural factors	- Organizational characteristics [24] - Resources and budget [24, 36] - job properties [27] - environmental changes [24] - Job characteristics [27]

## Discussion

The present study was conducted to investigate the reasons for resistance to change in nursing as an integrated review of various studies. In this review; three main categories (individual factors, interpersonal factors, and organizational factors), six sub-categories, and thirty-seven codes were identified.

What is clear to us is that change in improving patient outcomes is common and important in the current healthcare systems [40]. The process of change is an inevitable issue in healthcare, so understanding the benefits of change for patients is most likely to be successful when caregivers have the opportunity to influence change. Making changes can be challenging because they conflict with basic human needs for a sustainable environment [41]. Although changes in clinical environments are inevitable, resistance to them for various reasons; can be created.

Based on the findings of the present study, *individual factors*; It is one of the factors that can be based on the individual attitude and understanding and personality characteristics of nurses. Attitudes toward an impending change may be positive, negative, or neutral. Resistance to change in nursing is probably based on negative and defensive feelings toward change such as fear, uncertainty, doubt, disappointment, mistrust, confusion, and anger [42]. The findings of Amarantou’s study (2018) also confirm that resistance to change is indirectly influenced by individual’s emotions and personality characteristics [7]. Tendency to pessimism in employees is one of the personality characteristics that causes negative attitudes and perceptions toward change. The use of this defense mechanism is adopted unintentionally when danger occurs in order to reduce stress. The tendency to pessimism is directly related to a person’s personality and reflects a negative perception of human behavior and is characterized by pessimistic behavior and the inability to establish appropriate interpersonal relationships [43]. In the current study, individual personality characteristics indicate how pessimistic employees are toward change. Persons who have high levels of the above personality traits are more likely to experience negative emotional reactions, deny changes, show a judgmental and negative attitude towards change, and believe that the effect of implemented changes will be unfavorable [7].

All personnel in an organization does not react equally to ongoing changes in their organization [37]. A feeling of insecurity, doubt, and on the other hand, low motivation in implementing change [36], with a lack of trust and negative belief in change [26, 27], and a lack of readiness to accept change [35] seeks resistance to change. Individuals with conservative personality traits and low flexibility to change can also make this process more difficult [26]. Changes in the structure or design of organizations as a result of the introduction of new technologies are likely to lead to changes in work roles and increased feelings of uncertainty and insecurity among personnel. Job insecurity may cause personals to resist proposed changes [44]. When personnel are satisfied with their current position in the organization, they may become increasingly anxious about future changes because they fear that intrinsic rewards and well-being will be negatively affected. Consequently, when individuals feel that their well-being is threatened, they try to protect it and resist possible changes [45].

Among other effective factors that can cause RtC in nursing is *interpersonal factors* of employees. Studies show that communication barriers in the organization ultimately affect the implementation, quality, and sustainability of change [9]. Employees’ job perception includes rewards and inner satisfaction that they receive from their jobs and interactions with their colleagues [46], and the positive Colleagues’ opinion are indirectly effective in the resistance behavior of personnel and reducing resistance to change [47]. The quality of communication between employees is related both to the information before the implementation of the change and to the quality of the information during the implementation of the change [48]. This factor also refers to the overall quality of communication within the organization, and studies also indicate that poor communication is related to uncertainty in change and often magnifies the negative aspects of change and creates resistance to it. Also, inadequate cross-functional and vertical communication during the stage of change implementation, makes personnel more reluctant to the proposed changes, since they are less informed. So, communication quality will be negatively associated with attitude towards change, disposition towards change and anticipated impact of change [7, 49].

The third factor of resistance to change in nursing, is the *organizational factors* which are in three sub-categories; management factors, organizational values, and structural factors are placed. As mentioned, accepting change at the core of nursing and health care is a challenge because nurses are not only inflexible but also adept at strengthening the existing [24]. Therefore, changes in healthcare environments require effective managers who can implement change strategies to improve patient outcomes [40].

The effect of RtC can strengthen the negative effect on organizational effectiveness and organizational commitment, and the lack of leadership support will amplify with time. In this regard, it seems that managers supporting change in the organization can play an important role in improving resistance [5]. The results of the studies show that a key obstacle to the implementation of change is the culture reported by managers to change [6], that lack of proper education and guidance is one of the reasons for this [35], so it seems that the use of appropriate communication; education; feedback, and self-evaluation can be considered a suitable solution to overcome the resistance [6, 50]. In general, if the information provided about the change is timely, valid, informative, and sufficient, a more positive evaluation of the change will emerge in the individual [51]. The tendency to amplify the status quo and the difficulty of change application besides the lack of organizational support can cause resistance to change in nursing [35]. The lack of participatory management and not being involved in the change process can be considered a factor in the failure of change [12, 27]. Employee’s participation in decision-making; Responsibility and ownership of making changes amplify and can be effective in reducing resistance [52]. Low levels of participation and fear of job loss occur as a result of negative feelings towards change [53]. It is important to understand cultural change as involving strategic change, which consists of changing an individual’s mind and behavior. How the culture change for each individual is evoked also has an important impact on the result and the consequences for each person [54]. All noteworthy organizational changes require a few level of corporate culture alter. In spite of the fact that culture alter is essential for making and fortifying organizational change, our position is that making fundamental auxiliary changes may serve as the introductory intercession for changing culture [55].

Organizational culture is characterized as a set of anticipated behaviors that are for the most part supported inside the group [56], can play a significant role in RtC. The evidence indicates that to be more successful in the process of change resulting from the implementation of organizational culture, all nurses must be involved in this process from the beginning, otherwise, the employees will feel unappreciated and not involved, and resistance to change will be an unexpected result, and organizational commitment will be reduced [36, 57, 58]. By improving the understanding of the change process, nurses as change agents can meet the challenge of managing change in their clinical environment [40]. As mentioned; human resources education and amplifying proper communication is among the effective tools to overcome the resistance resulting from the organizational culture [6]. The nature of the relationship between employees and management, if the pessimism that employees express towards management to change, will mean that they will question the real motivations for implementing the change [43]. Employees who feel their managers are trustworthy, supportive, inspiring, and can better deal with change; will be more effective in dealing with resistance to change. Therefore, if there is a good relationship between the leadership and the organization’s members, it is expected to see less resistance to change [7].

Organizational values including organizational culture, negative organizational perception, and conflict with organizational identity also play a fundamental role to cause nurses’ resistance to change. Understanding organizational orientations may hinder the adoption of new evidence-based programs and practices [26]. Also, changes are frequently in conflict with the organizational identity, which causes an unpleasant impression on individual, and this leads to the distortion of the intended purpose of the change and puts the organizational identity at risk [28]. Change management starts before any change action is implemented and continues with an understanding of the culture and environmental context in which the change is to be implemented. So, it is important to ensure that change is not just implemented, but that employees and other stakeholders are ready for it [24].

Structural factors such as organizational characteristics, resources and budget, job characteristics, and environmental changes, along with other organizational factors, are effective factors in creating resistance to change in health care workers. Higgs and Rowland (2010) emphasize factors such as environmental changes, organizational characteristics, resources, and budgets as broad factors affecting the change process [36, 59]. Organizational changes are carried out with the aim of changing the way care is provided [60], one of these changes is related to job characteristics and employees with changes such as moving workplaces, creating new units, merging with existing units, and recruitment of new employee [61]. Based on this, the key strategies for change management should be focused on the need for sensitivity to organizational culture and characteristics [24].

Organizational change can be called a stressful factor in the work environment [62], but although these changes can lead to mental and physical stress among the healthcare team, providing support and positive organizational resources, such as job support and control, may help reduce nurses’ burnout and RtC. Studies also indicate that; when change-related stressors are high, nurses who report high levels of manager’s support; report lower levels of organizational support, and lower levels of cynicism toward workplace change than employees; who report low levels of organizational support [63]. So, changes in nursing work cause a high workload and increase in administrative stress, which ultimately leads to an increase in pessimism among them regarding the change. Trying to control the job in the organizational structure is necessary to deal with the increase in workload, and reduce pessimism and resistance to change [64].

Finally, based on the results obtained from selected studies, due to the nature of the nursing profession on the one hand and the occurrence of rapid and large changes in clinical environments and care organizations on other hand, several factors can cause resistance to these changes and affect the care and safety of patients. This resistance is influenced by three important factors, individual, interpersonal, and organizational factors. The effects of these factors, can directly and indirectly, affect the proper care of patients. Therefore, paying attention to these factors to improve them with education, improving communication, efficient and collaborative management, understanding organizational values, and developing organizational structure can reduce resistance to changes in patient care environments.

## Conclusion

Changes in the nursing environment are an integral part of nursing practice. The findings of this integrated review confirm the complexity and multifaceted nature of these resistances. A set of individual, interpersonal, and organizational factors in nurses leads to resistance to change and is considered an important challenge in nursing care. Knowing these factors can help reduce resistance and improve the quality of nursing care. So nursing managers and decision makers should pay special attention to this in order to make changes. So that, nurses can provide safe and qualified care for their clients and improve the level of health and satisfaction of patients.

### Limitations

The limitations of this study include: not searching for articles in languages other than English and Persian, so our search strategies may have under-represented studies in other languages, such as Spanish and Portuguese.

## Data Availability

The datasets used and/or analyzed during the current study are available from the corresponding author upon reasonable request. All requests relating to data should be addressed to rozitacheraghi@gmail.com.
